# Effect of exogenous glucoamylase inclusion on in vitro fermentation and growth performance of feedlot steers fed a dry-rolled corn-based diet^1^

**DOI:** 10.1093/tas/txab082

**Published:** 2021-05-10

**Authors:** Alejandro M Pittaluga, Shukun Yu, Wenting Li, Josh C McCann

**Affiliations:** 1 Department of Animal Sciences, University of Illinois, Urbana, IL 61801, USA; 2 DuPont Nutrition & Biosciences ApS, IFF, Brabrand 8220, Denmark; 3 Danisco Animal Nutrition, Wilmington, DE 19803, USA

**Keywords:** dry-rolled corn, exogenous glucoamylase, feedlot, in vitro digestion

## Abstract

The objective was to evaluate the effect of adding an exogenous glucoamylase (GA) enzyme from the fungus *Trichoderma reesei* on in vitro fermentation, growth performance, and carcass characteristics of feedlot steers fed a dry-rolled corn (DRC)-based diet. Experiment 1 evaluated three levels of added enzyme (0, 0.24, and 0.72 GA enzyme units) and two corn particle sizes (CPS; 2 and 4 mm) in a factorial arrangement using a 7 h in vitro batch culture fermentation. Addition of GA increased (*P* < 0.01) in vitro dry matter disappearance by 13% and decreased final pH (*P* < 0.01). Molar proportion of propionate increased with GA inclusion (*P* < 0.01). A smaller CPS increased (*P* < 0.01) in vitro dry matter disappearance and total volatile fatty acid and decreased final pH (*P* < 0.01). A smaller CPS also decreased (*P* < 0.01) the molar proportion of acetate and increased (*P* < 0.01) the molar proportion of butyrate. In experiment 2, Angus × Simmental steers (*n* = 105; initial body weight [BW] = 329 ± 38 kg) were used to evaluate the inclusion of an exogenous GA on growth performance and carcass characteristics. Steers were fed a basal diet consisting of 60% DRC, 17.5% modified distillers grains with solubles, 12.5% corn silage, and 10% dry supplement on a dry matter basis for 136 d. Steers were blocked by weight and allotted to pens. Pens were randomly assigned to one of three treatments (5 pens/treatment): diet with no GA (CON), low inclusion of GA (122 enzyme units/kg dry matter [DM]; LGA), or high inclusion of GA (183 enzyme units/kg DM; HGA). Inclusion of GA did not affect (*P* ≥ 0.23) final BW, dry matter intake (DMI), or average daily gain (ADG) for the 136-d feeding period. Feed conversion was affected (*P* = 0.02) by treatment with steers fed HGA having ~8% greater G:F compared with LGA and CON. Treatment did not affect (*P* = 0.32) fecal starch. Inclusion of GA did not affect (*P* ≥ 0.19) carcass traits including hot carcass weight, 12th rib fat thickness, yield grade, longissimus muscle area, or marbling score. Overall, results suggest inclusion of exogenous GA enzyme increased in vitro dry matter disappearance in batch culture and improved feed conversion in steers fed 183 enzyme units/kg DM during the finishing phase.

## INTRODUCTION

Finishing diets are composed of about 45% to 55% starch based on typical dietary corn inclusion (Samuelson et al., 2016). While the rumen is the primary site of starch digestion, it is highly dependent on grain source, processing method, and degree of grain processing (Owens et al., 1997). Steam flaking corn usually improves cattle performance due to an increase of starch digestibility compared with simpler processing methods like dry-rolling (Zinn et al., 2002). However, many feedlot cattle are fed dry-rolled corn (DRC)-based diets due to lower processing costs and geographical location. Dry-rolled corn-based diets have 10% lower feed conversion than steam-flaked corn-based diets in part due to decreased starch digestibility (Owens et al., 1997). Exogenous α-amylases (often abbreviated as amylases, enzyme commission 3.2.1.1) have been proposed as an effective mechanism to improve ruminal starch digestion (Mora-Jaimes et al., 2002; Rojo et al., 2005) and increase milk production and efficiency of dairy cattle (Tricarico et al., 2005; Klingerman et al., 2009; Gencoglu et al., 2010). While the impact of exogenous amylases in growth performance has been evaluated with limited success in feedlot cattle, glucoamylase (GA, syn. amyloglucosidase, enzyme commission 3.2.1.3) research focused on digestion and fermentation measures rather than beef cattle performance. Importantly, the enzymatic activity of GA yields a single glucose molecule that can be directly used by rumen microbiota. In contrast, amylases yield predominantly maltodextrins and maltose that often need to be hydrolyzed to glucose before entering cellular metabolism (Norouzian et al., 2006). Our objectives were to evaluate the effect of adding different doses of an exogenous GA from the fungus *Trichoderma reesei* on in vitro corn digestibility and on growth performance and carcass characteristics of feedlot steers fed a DRC-based finishing diet.

## MATERIALS AND METHODS

All experimental procedures were approved by the Institutional Animal Care and Use committee of the University of Illinois (IACUC #16103 and #15008) and followed the guidelines recommended in the Guide for the Care and Use of Agricultural Animal in Agricultural Research and Teaching (FASS, 2010). Glucoamylase produced by *T. reesei* fermentation was provided by DuPont Nutrition & Biosciences (Rochester, NY) with the activity strength of approximately 500 U/g.

### Experiment 1

A completely randomized design with a 2 × 3 factorial arrangement of treatments was used to evaluate an interaction of GA with corn particle size (CPS) in a batch in vitro fermentation. Treatments included three doses of exogenous GA (0, 0.24, and 0.72 enzyme units) and two CPSs (2 and 4 mm). Inclusion of 0 enzyme units served as the control. Each treatment combination was run in triplicate and the entire experiment was replicated three times.

One liter of ruminal fluid was collected and pooled from two rumen fistulated crossbred Angus steers being fed a DRC-based feedlot diet. Rumen contents were squeezed through four layers of cheesecloth into a prewarmed thermos. Then fluid was transferred into separatory funnels, placed in 39 °C water bath and mixed with warmed McDougall’s buffer (McDougall, 1948). The ratio of buffer to rumen fluid was 4:1 (v/v). The basic in vitro incubation procedure was carried out according to Tilley and Terry (1963). Briefly, corn grain substrate was ground using two different screen sizes (2 and 4 mm), weighed (0.5 g), and placed in 50 mL polypropylene fermentation tubes fitted with a rubber stopper and one-way valve. A volume of 30 mL of the inoculum was added to tubes. The GA enzyme was diluted in distilled water at a 10% concentration and added (<100 µL) to the tubes. No liquid was added to the tubes containing 0 enzyme units. After being flushed with CO_2_, tubes were placed in water bath at 39 °C. All the tubes were mixed initially and every 2 h. Fermentation was terminated after a 7-h incubation period.

At the end of the incubation, pH was measured using a combination electrode (Thermo Fisher Scientific, Waltham, MA). Then the fermentation was terminated by adding 0.5 mL of 0.2 N HCl to each tube and placing the contents in the freezer. Tubes were centrifuged at 1,833 × *g* for 15 min at 4 °C. Before decanting the supernatant liquid, 3 mL were preserved at −20 °C for volatile fatty acid and ammonia-N analysis. All remaining solids were filtered with Whatman filter papers 541 (GE Healthcare Life Science, Chicago, IL) and dried at 105 °C for approximately 4 h. In vitro dry matter disappearance was calculated as following: −blank))/initial dry sample wt] −blank))/initial dry sample wt].

Volatile fatty acids were determined according to Erwin et al. (1961) by gas chromatography (HP1850 series gas chromatography Hewlett-Packard, Wilmington, DE) on a glass column. Ammonia-N was determined by colorimetric procedures described by Broderick and Kang (1980) using a UV–visible spectrophotometer at 630 nm absorbance (Perkin-Elmer Model 2380, Waltham, MA).

The MIXED procedure of SAS 9.4 (SAS Inst. Inc., Cary, NC) was used for all statistical analysis. The experimental unit was the individual tube. The model included the fixed effects of CPS, exogenous GA dose, and their interactions in addition to the random effect of experimental run. Significance was declared at *P* < 0.05.

### Experiment 2

To evaluate the effect of different inclusion levels of GA on cattle growth performance, 105 Angus × Simmental steers (initial body weight [BW] = 329 ± 38 kg) were fed for 136 d at the Beef and Sheep Field Research Laboratory in Urbana, IL. The experiment was conducted as a randomized block design. Steers were blocked by BW, stratified by sire, and assigned to 15 pens with 7 steers in each pen. Pens were assigned randomly to one of three treatments. Treatments included a finishing diet with no enzyme addition (negative control; CON), the finishing diet with a low dose (122 enzyme units/kg dry matter [DM]) of exogenous GA (LGA; DuPont Industrial Bioscience, Wilmington, DE), and the finishing diet with a high dose (183 enzyme units/kg DM) of the exogenous GA (HGA). Original targeted doses for LGA and HGA were 115 and 173 GA U/kg DM, respectively. The finishing diets (Table 1) were offered ad libitum in GrowSafe (GrowSafe Systems Ltd, Calgary, AB, Canada) bunks for the duration of the study. Pens were fed once daily in the morning at 0900 h until d 100 where feed was delivered twice per day. The liquid GA enzyme solution was stored at −4 °C until feed mixing, then diluted in water (~2 L) and mixed with corn on the feed truck. A colored water solution was used for the CON diet to mimic the color of the exogenous GA and ensure feeders were blinded to treatment.

**Table 1.** Ingredients and analyzed nutrient content of the basal diet

Ingredient, % of DM	Basal diet
DRC	60.0
Modified distillers grains with solubles	17.5
Corn silage	12.5
Supplement premix	
Ground corn	7.435
Limestone	1.708
Urea	0.650
Trace mineral premix^*a*^	0.098
Rumensin 90^*b*^	0.017
Tylosin 40^*b*^	0.011
Fat	0.081
Analyzed composition, % of DM	
Dry matter	66.1
Organic matter	96.4
Crude protein	13.7
Neutral detergent fiber	14.5
Acid detergent fiber	6.3
Ether extract	4.7
Calculated composition, % of DM^*c*^	
NE_m_, Mcal/kg	2.12
NE_g_, Mcal/kg	1.46

^*a*^8.5% Ca, 5% Mg, 7.6% K, 6.7% Cl, 10% S, 0.5% Cu, 2% Fe, 3% Mn, 3% Zn, 278 mg/kg Co, 250 mg/kg I, 150 mg/kg Se, 2,205 KIU/kg Vit A, 662.5 KIU/kg Vit D, and 22,047.5 IU/kg Vit E.

^*b*^Elanco Animal Health, Greenfield, IN.

^*c*^Composition was calculated based on NASEM (2016).

Steers were weighed on 2 consecutive days at the beginning and end of the experiment. At the beginning of the experiment, all steers were implanted with Component TE-IS (Elanco Animal Health, Indianapolis, IN) and then reimplanted with Component TE-S (Elanco Animal Health, Indianapolis, IN) on d 57. Steers were slaughtered at a commercial abattoir located 300 km from the feedlot. Hot carcass weight (HCW) was recorded immediately after slaughter. Individual camera carcass data and carcass characteristics were provided by Tyson Foods Inc. Yield grade was calculated according to USDA (1997).

Steers were housed in a covered barn in pens (4.88 × 4.88 m) with concrete slats covered with rubber matting. Cattle were provided with fresh water ad libitum. Each pen contained one GrowSafe bunk (0.91 × 0.53 × 0.38 m) that allowed only one steer to eat at a time. Intake data were removed on individual days if less than 85% of the consumed feed was assigned to steers or if the bank was empty for more than 12 h.

Feed ingredients samples were collected biweekly and frozen at −20 °C and composited at the end of the experiment. Composite samples were lyophilized and ground in a Wiley mill (Arthur H. Thomas, Philadelphia, PA). Ingredients were analyzed for dry matter (24 h at 105 °C), neutral detergent fiber (NDF) and acid detergent fiber (ADF; using Ankom Technology methods 5 and 6, respectively; Ankom 200 Fiber Analyzer, Ankom Technology), crude protein (CP; Leco TruMac, LECO Corporation, St. Joseph, MI), ether extract (EE, Ankom method 2; Ankom Technology), and ash (600 °C for 2 h; Thermolyte muffle oven Model F30420C; Thermo Scientific, Waltham, MA).

Fresh feces samples (~200 g) from 2 random steers/pen were collected on d 112 and d 135. Samples were frozen at −20 °C and analyzed at a commercial laboratory (Cumberland Valley Analytical Services, Waynesboro, Pennsylvania, PA) for fecal starch (FS) content. Total starch digestibility (TSD) was calculated from FS content using an equation reported by Owens et al. (2016), where $TSD\%=0.0102\times FS^{2}-0.3621\times FS+99.701$.

All statistical analyses were conducted using the MIXED procedure of SAS 9.4 (SAS Inst. Inc., Cary, NC) for a randomized block design. Pen was the experimental unit. The model was fitted with individual animal data and included the fixed effects of block and treatment as well as a random effect of pen nested within treatment (St-Pierre, 2007). Expected progeny differences (EPDs; mid-parent average) were used as a covariate to account for inherent genetic differences by selecting the most appropriate EPD for the corresponding response variable (Shike, 2018). Initial body weight was also used as a covariate for growth performance variables. One steer assigned to CON was removed from the study for reasons unrelated to treatment and was excluded from all analysis. Analysis of FS and predicted starch digestibility included a fixed effect of date and the treatment by date interaction in addition to the fixed effect of sire. Significance was declared at *P* < 0.05.

## RESULTS AND DISCUSSION

### Experiment 1

There were no interactions between dose and CPS (*P* ≥ 0.08) for IVDMD, final pH, ammonia concentration, or volatile fatty acids (VFAs; Table 2). Including the exogenous GA increased (*P* < 0.01) the IVDMD of ground corn by 13%. Final pH and ammonia concentration decreased (*P* < 0.01) with the addition of GA. Total VFA was not altered (*P* ≥ 0.11) by GA. Experimental GA inclusion did not affect (*P* = 0.43) the molar proportion of butyrate, but did increase (*P* < 0.01) the molar proportion of propionate.

**Table 2.** Effects of exogenous GA dose and CPS in a 7-h batch fermentation in experiment 1

	GA^*a*^				CPS^*b*^			*P* value		
Item	0	0.24	0.72	SEM	2	4	SEM	GA	CPS	GA × CPS
IVDMD^*c*^, %DM	43.8	51.8	51.8	5.78	53.3	46.3	5.76	<0.01	<0.01	0.10
Final pH^*d*^	6.55	6.45	6.44	0.016	6.41	6.55	0.015	<0.01	<0.01	0.12
NH_3_-N^*e*^, mmol/L	5.87	5.42	4.99	0.324	4.89	5.96	0.311	<0.01	<0.01	0.22
Total VFA, mM	58.4	60.2	61.1	2.12	63.1	56.7	2.05	0.11	<0.01	0.08
VFA, molar %										
Acetate	45.6	45.1	45.3	0.4	45.1	45.6	0.4	0.07	<0.01	0.51
Propionate	38.2	39.0	39.0	1.8	38.8	38.7	1.8	<0.01	0.54	0.95
Butyrate	13.0	12.9	12.7	1.1	13.2	12.6	1.1	0.43	<0.01	0.74

^*a*^GA inclusion: 0 = no inclusion of GA; 0.24 = 0.24 units of GA enzyme; 0.72 = 0.72 units of GA enzyme.

^*b*^Corn particle size (2 and 4 mm).

^*c*^In vitro dry matter disappearance after 7 h incubation.

^*d*^Final pH of the fluid inside the tubes after 7 h incubation.

^*e*^Ammonia concentrations of the in vitro fluid after 7 h incubation.

An in vitro ruminal digestion of corn using amylase and GA from *Bacillus licheniformis* and *Aspergillus niger*, respectively, observed increases of 8% to 13% for DM disappearance but there was no effect of enzyme doses (Rojo et al., 2007). Likewise, in vitro DM disappearance of corn and sorghum grain increased when adding amylase after 6 and 12 h of incubation (Velasco, 2004; Crosby et al., 2012). In addition, exogenous GA from *A. niger* increased 24 h in vitro DM degradation when included in a substrate containing 30% and 15% ground corn and sorghum, respectively (Mendoza et al., 2013). Mixing ground corn with an amylase preparation 24 h before incubation improved in situ (Gutierrez et al., 2005) and in vivo (Rojo et al., 2005) ruminal degradation. Overall, the observed increase in IVDMD of the corn substrate agrees with existing literature.

Addition of the experimental GA decreased the final pH of the ruminal fluid in vitro likely due to increased DM disappearance, but did not affect total VFA concentrations. Comparable to the observed increase in the proportions of propionate, Rojo et al. (2005) also reported greater propionate when evaluating the in situ ruminal fermentation of a ground sorghum-based diet with 1.45 and 2.90 g/kg DM sorghum of an amylase from *B. licheniformis*; however, similar doses of a GA from *A. niger* quadratically decreased propionate. The more acidic environment due to greater DM degradation from GA inclusion may have contributed to greater propionate as a lower pH can shift VFA molar proportions toward propionate through both the succinate and acrylate pathways (Russell, 1998). In high-concentrate diets, in vitro experiments suggest the succinate pathway accounts for the majority of the glucose converted to propionate compared with the acrylate pathway (Baldwin et al., 1963). The greatest effect of GA inclusion in an in vitro system was observed at 0.24 enzyme units with no benefit of a higher inclusion in a 7-h batch fermentation.

Reducing CPS increased (*P* < 0.01) IVDMD and total VFA while reducing (*P* < 0.01) the final pH and ammonia concentrations. With smaller CPSs, the molar proportions of acetate decreased (*P* < 0.01) while butyrate increased (*P* < 0.01). Molar proportion of propionate was not altered (*P* = 0.54) by the substrate particle size. The greater disappearance of smaller granule corn particles is well established (Dhital et al., 2010); thus, a greater IVDMD was expected for the 2 mm CPS. Reducing the particle size of the ground corn increased substrate availability and surface area favoring enzymes of both exogenous and microbial origin. Considering no interactions were observed for CPS and exogenous GA, their effects can be considered independent and additive using this in vitro methodology.

### Experiment 2

#### Finishing growth performance

At the conclusion of the 136-d finishing phase, no differences were observed for final BW, average daily gain (ADG), or dry matter intake (DMI) (*P* ≥ 0.23) between dietary treatments (Table 3). However, G:F was affected (*P* = 0.02) by treatment with HGA being ~8% greater compared with LGA and CON.

**Table 3.** Effect of exogenous GA supplementation on performance of feedlot beef steers in experiment 2

	Treatment^*a*^				
Item^*b*^	CON	LGA	HGA	SEM	*P* value
Initial BW, kg	325	322	322	4.0	0.85
Final BW, kg	573	581	583	7.2	0.63
ADG, kg	1.78	1.84	1.85	0.055	0.62
DMI, kg	9.46	9.79	9.15	0.256	0.23
G:F	0.189	0.189	0.204	0.0045	0.02

^*a*^Treatments: CON = basal diet without GA; LGA = low GA inclusion (122 enzyme units/kg DM); HGA = high GA inclusion (183 enzyme units/kg DM).

^*b*^A 4% pencil shrink was applied to all live BW measures including G:F calculations.

Although the efficacy of exogenous GA has been evaluated in vitro, there is a dearth of literature on their effects in beef cattle. Lambs have been most commonly used to evaluate use of exogenous GA in vivo. An exogenous GA added at 1.5 mL enzyme protein/kg DM resulted in a 4% increase in total tract DM digestion in lambs fed diets composed of 30% and 15% of ground corn and sorghum grain, respectively (Mendoza et al., 2013). However, no effects on growth performance or feed conversion were observed in this metabolism trial that used 7 lambs/treatment (Mendoza et al., 2013). Including an exogenous GA in a diet with 70% ground sorghum 24 h before feeding did not affect total tract digestion in lambs, but protozoa counts, ruminal ammonia, and total VFA concentration were increased by GA inclusion (Rojo et al., 2005). While much of the early research on GA focused on digestion and fermentation measures, the studies were not well powered to draw conclusions based on growth performance (Rojo et al., 2005; Lee-Rangél et al., 2006, Mendoza et al., 2013).

Although GA and amylases have distinct modes of action, more research has evaluated the use of amylases in feedlot cattle. Incorporating two different doses of an amylase preparation from *B. licheniformi*s in a DRC or steam-flaked corn-based diets did not affect ADG or feed conversion of feedlot steers (DiLorenzo et al., 2011). Similarly, Tricarico et al. (2007) observed no differences in the overall finishing performance of beef heifers when including an amylase preparation from *Aspergillus oryzae* at 0, 580, or 1,160 dextrinizing units/kg DM in a cracked corn or high moisture corn-based diet.

Alternatively, exogenous amylase preparations have been more extensively evaluated in diets fed to dairy cattle with multiple examples of a production-level benefits. Including an exogenous amylase preparation (300 kilo novo units/kg DM) from *Geobacillus stearothermophilus* in dairy cow diets increased apparent total tract OM digestion which likely drove improvements in milk production and fat corrected milk efficiency (Gencoglu et al., 2010). Milk production of dairy cows was also enhanced when an amylase preparation from *A. oryzae* was included in the diet (Tricarico et al., 2005; Klingerman et al., 2009). An exogenous amylase produced by *B. licheniformis* has also improved feed conversion in dairy cattle, which was related to differences in feed sorting behavior (Andreazzi et al., 2018). Nonetheless, Ferraretto et al. (2011) and Weiss et al. (2011) did not observe any benefit by supplementing exogenous amylase to dairy cows. An inconsistent response to exogenous enzymes should be expected in a production setting based on many factors affecting their individual efficacy including the composition of the basal diet, diversity of enzymatic preparations, enzyme stability, enzyme inclusion level, and wide variety of enzymes that exist (McAllister et al., 1999).

Fecal starch and the estimated TSD did not differ (*P* ≤ 0.25) between treatments and sampling dates of fecal collections (Table 4). There was no observed (*P* ≤ 0.25) treatment and date interactions for FS and TSD. Accordingly, adding exogenous amylases has typically not affected total tract starch digestion regardless of the grain content and processing method (Klingerman et al., 2009; Gencoglu et al., 2010; DiLorenzo et al., 2011; Weiss et al., 2011).

**Table 4.** Effect of exogenous GA supplementation on fecal starch (FS) and total starch digestibility (TSD) of feedlot steers in experiment 2

	Treatment^*a*^				*P* value^*b*^		
Item	CON	LGA	HGA	SEM	Trt	Date	Trt × Date
FS^*c*^, %DM	28.0	22.3	22.1	3.20	0.27	0.65	0.25
TSD^*d*^, %	80.3	85.6	85.9	2.84	0.25	0.56	0.29

^*a*^Treatments: CON = basal diet without GA; LGA = low GA inclusion (122 enzyme units/kg DM); HGA = high GA inclusion (183 enzyme units/kg DM).

^*b*^Trt = main effect of treatment; Date = main effect of sample collection date; Trt × Date = interaction of treatment by date.

^*c*^Starch content in feces samples obtained in d 112 and d 135 expressed as % of fecal DM.

^*d*^Total tract starch digestibility estimated from the FS as suggested by Owens et al. (2016).

The obtained results concur with the proposed hypothesis that including the novel GA in a DRC-based diet will improve cattle performance. However, the mode of action of the exogenous GA responsible for the improvement in feed conversion are not clear. Despite data suggesting amylolytic activity of rumen microorganisms does not appear to limit starch digestibility (Hristov et al., 1999; DiLorenzo et al., 2011), exogenous amylase preparations can increase in vivo ruminal starch digestion (Rojo et al., 2005; Nozière et al., 2014). It is plausible that including the experimental GA generated numerical differences in FS and the estimated TSD sufficient to alter feed conversion. Furthermore, incremental levels of GA in the diet may affect DM digestibility by altering the digestion of other dietary components such as fiber. A greater release of maltodextrins from starch in the rumen as a result of exogenous α-amylase inclusion, has been postulated to promote the growth of nonamylolytic microorganisms and thus modify the microbial metabolism or population (Tricarico et al., 2008). Nonamylolytic bacteria are capable of rapid growth in a maltodextrins-rich media, but exhibit a reduced growth rate in starch-rich media (Cotta, 1992; Tricarico et al., 2008). Moreover, exogenous amylase inclusion have also increased total tract NDF digestion (Klingerman et al., 2009; Gencoglu et al., 2010; Weiss et al., 2011), but NDF digestibility was not measured in the present study.

Another mode of action that could translate to feed conversion is increased ruminal propionate. This is supported by the greater molar proportions of propionate in experiment 1 and observations in dairy cattle (Nozière et al., 2014). Greater propionate has been observed in a cows ruminally infused with glucose (Wu et al., 1994), which translated to increased milk protein percentage and may be related to greater feed efficiency observed in the present study. Increasing ruminal propionate is usually associated with a decline in methane production, which results in greater energy availability for the animal (Bergen and Bates, 1984). Energy losses from the diet decrease when reducing equivalents generated during ruminal fermentation are diverted away from methanogenesis, and alternatively disposed in more advantageous electron-sink pathways for the animal, such as propionate synthesis (Newbold et al., 2005).

#### Carcass characteristics

Carcass characteristics are summarized in Table 5. Longissimus muscle (LM) area, HCW, 12th rib fat, kidney–pelvic–heart fat (KPH) were not different (*P* ≥ 0.19) among treatments. Likewise, yield grade and marbling score were not different (*P* ≥ 0.76) among treatments. Available data on the effect of exogenous amylases in carcass characteristics of beef cattle are scarce and are not in agreement with the observed results of this study. Adding 950 dextrinizing units/kg of DM of an exogenous amylase from *A. oryzae* to the diet of feedlot steers fed a steam-flaked corn-based diet increased LM area regardless of the roughage source (Tricarico et al., 2007). When feeding exogenous amylases to feedlot heifers on a DRC or high moisture corn-based diet, HCW and LM area quadratically increased, but yield grade quadratically decreased across both corn processing methods (Tricarico et al., 2007).

**Table 5.** Effect of exogenous GA supplementation on carcass characteristics of feedlot steers in experiment 2

	Treatment^*a*^				
Item	CON	LGA	HGA	SEM	*P* value
HCW^*b*^, kg	364	370	370	4.4	0.58
Dress, %	64.2	64.1	63.9	0.26	0.62
Yield grade	2.8	2.8	2.9	0.07	0.84
LM area, cm^2^	90.9	90.1	92.6	1.26	0.37
12th rib fat thickness, cm	1.29	1.33	1.42	0.049	0.19
Marbling score^*c*^	449	442	455	12.5	0.76
KPH^*d*^, %	2.00	1.95	1.99	0.030	0.45

^*a*^Treatments: CON = basal diet without GA; LGA = low GA inclusion (122 enzyme units/kg DM); HGA = high GA inclusion (183 enzyme units/kg DM).

^*b*^Hot carcass weight.

^*c*^Marbling score; scale: 400–499 = small, 500–599 = modest, 600–699 = moderate, 700–799 = slightly abundant, 800–899 = moderately abundant.

^*d*^Kidney–pelvic–heart fat.

## CONCLUSION

With low and high inclusion of GA, in vitro DM disappearance of corn increased, as well as the molar proportions of propionate during a 7 h incubation period. A high dose of an experimental GA in a DRC-based diet improved the feed conversion without any negative effect on carcass characteristics. Including an exogenous GA at the higher dosage in finishing diets can be an effective mechanism to improve feed conversion in feedlot cattle. Further research is needed to determine the precise mechanisms and refine GA dosage for maximum benefit to feedlot cattle performance fed DRC-based diets.

*Conflict of interest statement*. A.M.P. has no conflict of interest to declare. W.L. and S.Y. are employed by DuPont Nutrition and Biosciences, the company that provided funding for these experiments. J.C.M. has provided consulting services to DuPont Nutrition and Biosciences.
